# Decreased respiratory complexity during apnea in acute respiratory distress syndrome

**DOI:** 10.1186/cc12031

**Published:** 2013-03-19

**Authors:** A Batchinsky, C Necsoiu, T Langer, V Vecchi, W Baker, J Salinas, L Cancio

**Affiliations:** 1 US Army Institute of Surgical Research, Fort Sam Houston, TX, USA; 2 University of Milan, Italy

## Introduction

Respiratory complexity (RC) as assessed by sample entropy (SampEn) is lower in patients failing spontaneous breathing trials. We evaluated the role of RC in monitoring subjects with severe acute respiratory distress syndrome (ARDS) treated with the Cardiohelp (Maquet, Rastatt, Germany) extracorporeal life-support system (ECLS). We hypothesized that RC is reduced during apnea.

## Methods

Six sheep sedated with midazolam were connected to the Cardiohelp via a 23F Avalon catheter. Blood flow was ~2 l/minute and FiO_2 _was 0.5. Sheep were on CPAP of 8 cmH_2_O with FiO_2 _of 1 via tracheostomy. After ~6 hours of ECLS, ARDS was induced by injection of 0.1 ml/kg oleic acid. Apneas (defined as >60% reduction in minute ventilation) developed both before (Preinjury) and after ARDS (Postinjury). Heart rate (HR), mean arterial pressure (MAP), respiratory rate (RR) and tidal volume (TV) were recorded, and the PaO_2_:FiO_2 _ratio (PFR) calculated corresponding to sections of respiratory pressure waveforms containing 200 breaths during Baseline, during Preinjury and Postinjury apnea periods. Similar periods were selected before and after apneas. Inter-breath interval (IBI) and its deviation, SampEn, percentage of forbidden words (FW), bit per-word entropy (BPWEn), symbol distribution entropy (DisNEn), and stationarity (StatAv) were calculated using software. Statistics by one-way ANOVA with adjustment for multiple comparisons.

## Results

See Table 1. SampEn was associated with PFR (*r*^2 ^= 0.31, *P <*0.001).

**Table 1:** 

		Preinjury	Preinjury	Preinjury	Postinjury	Postinjury	Postinjury
	**BL**	**pre-apnea**	**apnea**	**post-apnea**	**pre-apnea**	**apnea**	**post-apnea**
RR (breaths/minute)	22 ± 2	22 ± 3	17 ± 2	23 ± 4	24 ± 7	5 ± 1*	25 ± 7
TV (ml)	388 ± 31	217 ± 32	145 ± 14	205 ± 21	211 ± 51	116 ± 30*	229 ± 40
HR (beats/minute)	156 ± 15	153 ± 21	152 ± 21	166 ± 21	162 ± 22	165 ± 21	151 ± 16
MAP (mmHg)	102 ± 4.12	102 ± 6.13	103 ± 6.91	104 ± 6.08	106 ± 3.76	108 ± 3.86	111 ± 5.69
PFR (unitless)	435 ± 24.80	417 ± 48.06	354 ± 49.52	429 ± 37.87	139 ± 50.90*	92 ± 30.43*^#^	91 ± 27.25*
IBI (ms)	2,765.1 ± 230.5	3,149.8 ± 378.0	5,151.7 ± 467.7	3,404.7 ± 663.0	2,253.2 ± 427.0	10,334.0 ± 1,739.2*^#^	2,162.0 ± 387.6
IBI Dev (ms)	771.8 ± 117.49	1,630.0 ± 230.64	4,505.7 ± 785.34	1,598.3 ± 292.45	1,404.8 ± 445.04	25,065.0 ± 11,480.37*^#^	844.6 ± 236.65
SampEn (unitless)	1.7 ± 0.12	1.6 ± 0.16	1.3 ± 0.13	1.6 ± 0.11	1.2 ± 0.14	0.5 ± 0.25*^#^	1.3 ± 0.20
FW (%)	29.9 ± 4.60	41.5 ± 7.43	41.8 ± 10.15	34.5 ± 6.10	44.0 ± 8.80	62.2 ± 4.16*	33.6 ± 5.26
BPwEn (unitless)	4.9 ± 0.18	4.3 ± 0.27	4.1 ± 0.37	4.6 ± 0.22	4.1 ± 0.42	2.7 ± 0.38*^#^	4.4 ± 0.24
DisNEn (unitless)	0.8 ± 0.03	0.7 ± 0.04	0.7 ± 0.06	0.8 ± 0.05	0.7 ± 0.07	0.4 ± 0.06*^#^	0.7 ± 0.04
StatAv (unitless)	0.42 ± 0.06	0.37 ± 0.09	0.37 ± 0.04	0.41 ± 0.10	0.31 ± 0.05	0.51 ± 0.07	0.42 ± 0.08

*Significant difference versus Baseline. ^#^Significant difference between Preinjury and Postinjury apnea.

## Conclusion

Respiratory complexity during apnea and ECLS remains unchanged in a healthy state but decreases after ARDS. It has a significant association with PFR and may serve as an index of injury severity.

